# The Epworth Sleepiness Scale in the Assessment of Sleep Disturbance in Veterans with Tinnitus

**DOI:** 10.1155/2015/429469

**Published:** 2015-01-08

**Authors:** Yuan F. Liu, Jinwei Hu, Matthew Streelman, O'neil W. Guthrie

**Affiliations:** ^1^Department of Otolaryngology-Head and Neck Surgery, Loma Linda University Medical Center, 11234 Anderson Street, Loma Linda, CA 92354, USA; ^2^Loma Linda University School of Medicine, 11175 Campus Street, Loma Linda, CA 92350, USA; ^3^Loma Linda Veterans Affairs Medical Center, 11201 Benton Street, Loma Linda, CA 92357, USA

## Abstract

*Purpose*. Tinnitus and sleep disturbance are prevalent in veterans, and a better understanding of their relationship can help with tinnitus treatment. *Materials and Methods*. Retrospective chart review of 94 veterans seen in audiology clinic between 2010 and 2013 is presented. *Results*. The mean age was 62 years, and 93 of 94 veterans were males. The majority (96%) had hearing loss. The positive predictive value of the ESS for sleep disorder was 97% and the negative predictive value was 100%. Veterans with a Tinnitus Handicap Inventory (THI) score ≥38 had significantly higher Epworth Sleepiness Scale (ESS) scores compared to those with THI score <38 (*P* = 0.006). The former had a significantly higher incidence of PTSD, anxiety, and sleep disorder. A subgroup of patients had normal sleep despite rising THI scores. Bilateral tinnitus, vertigo, and anxiety were found to be predictors of sleep disturbance. *Conclusions*. The ESS can be used as a tool in the initial assessment of sleep disorders in veterans with tinnitus. Higher tinnitus handicap severity is significantly associated with greater sleep disturbance. Optimal management of tinnitus may require concomitant treatment of sleep disorder, PTSD, anxiety, and depression.

## 1. Introduction

Tinnitus, defined as the perception of sound without an external source, affects an estimated 35 to 50 million Americans or 5 to 15% of the general population [1–5]. Although the majority of those affected by tinnitus habituate to the condition and do not seek treatment, 10–20% experience tinnitus as a severe handicap [6].

Those who live with tinnitus may be burdened by comorbid stressors such as sleep disorders, depression, anxiety disorder, and suicidal ideation [7–9]. These conditions negatively impact many aspects of daily life, causing impairments in work and memory and reducing quality of life [7, 8, 10].

Sleep disturbance is one of the most common comorbidities associated with tinnitus [11–13]. The prevalence of sleep disorders in patients with tinnitus varies from 25 to 77%, with almost 50% reporting insomnia [6, 14–16]. Hébert and Carrier found that patients suffering from tinnitus reported greater sleep difficulties compared to controls, specifically in sleep efficiency and sleep quality [17]. Using questionnaires, other studies have found a higher prevalence of sleep complaints in tinnitus sufferers compared to the general population [13, 18]. It has also been shown that sleep disturbance strongly predicts lower tinnitus tolerance, while successful treatment of tinnitus results in fewer sleep complaints [11, 17, 19–21].

There is no definitive explanation of how tinnitus may lead to sleep disturbance. It has been postulated that when environmental noise wanes at night, tinnitus awareness may rise with the initiation of unhelpful thoughts, mood changes, and physical reactions, thereby initiating a cycle of anxiety, arousal, and distress [22]. Whatever the mechanism, it is important to note that insomnia is associated with functional impairment and decreased quality of life, especially among subjects with tinnitus who are older than 50 years of age [2, 11, 14, 23].

There are currently more than 20 million US veterans, a predominantly male, aging population with a history of noise exposure that is served by the Veterans Health Administration [24]. This cohort is especially prone to developing tinnitus, which has an increasing prevalence with age, and affects men more commonly than women [4, 25–27].

With the aid of the Epworth Sleepiness Scale (ESS), Tinnitus Handicap Inventory (THI), Tinnitus and Hearing Survey (THS), and Tinnitus Problem Checklist (TPC), we sought to address the following questions: can the ESS be used in the initial assessment of sleep disturbance in veterans with tinnitus? If so, among veterans with different degrees of tinnitus handicap severity, is there a difference in sleep disturbance? Are there any differences in demographics, hearing profiles, or psychiatric comorbidities that can distinguish veterans with different degrees of tinnitus? Can any characteristics of veterans with tinnitus help predict sleep disturbance?

## 2. Materials and Methods

### 2.1. Data Gathering

A retrospective chart review was conducted at the Veterans Affairs Medical Center (VAMC) in Loma Linda, CA, on patients with tinnitus complaints. The Loma Linda VAMC serves about 67,000 veterans living in the San Bernardino and Riverside Counties. Audiologic, otologic, and psychiatric data were retrieved from the computerized patient record system (CPRS). Charts for 117 patients were reviewed and 94 patients who had at least a completed Tinnitus Handicap Inventory and pure-tone audiometry data were included in the final analyses. These charts were from patients who visited the Loma Linda VAMC audiology clinic between 2010 and 2013. The data collected from CPRS included ICD-9 codes along with qualitative (e.g., type, severity, and degree) and quantitative (e.g., parametric results from diagnostic and screening tests) data when available. All procedures were reviewed and approved by the Institutional Review Board at the Loma Linda VAMC.

### 2.2. Epworth Sleepiness Scale (ESS)

The ESS is a standardized tool used to measure daytime sleepiness. It contains 8 questions, each scoring 0–3 with increasing number signifying higher chance of “dozing” while engaged in specific activities of daily life. A score of less than 10 is generally considered clinically normal [28–30]. The ESS was developed in 1991, was modified in 1997, and has become the most frequently used method worldwide for assessing daytime sleepiness due to its reliability, consistency, and ease of use [28, 31, 32].

### 2.3. Tinnitus Handicap Inventory (THI)

The THI is a survey containing 25 questions. Each question is worth up to 4 points, with 4 for “yes,” 2 for “sometimes,” and 0 for “no,” for a total of 100 points. A score of 0–16 denotes no handicap from tinnitus, 18–36 denotes mild handicap, and 38–56 denotes moderate handicap, and higher scores denote severe handicap. The questions can be divided into three categories which contribute to three subscales: functional (11 questions, 48 points), emotional (9 questions, 32 points), and catastrophic (5 questions, 20 points). The functional score reflects the effect of tinnitus on mental, social, occupational, and physical functioning. The emotional score reflects affective response to tinnitus. The catastrophic score reflects desperation, inability to escape, perception of having a terrible disease, lack of control, and inability to cope with tinnitus. The THI has been validated for being a robust tool for measuring the effect of tinnitus on daily life, with a score of 38 or higher suggesting significant tinnitus requiring intervention [33–36].

### 2.4. Tinnitus and Hearing Survey (THS)

The THS contains 10 questions; each scored 0–4 for increasing tinnitus, hearing, and/or sound intolerance severity. Four questions are for tinnitus, 4 questions are for hearing loss, and 1 question is for hyperacusis. A final, unscored question is answered yes or no regarding severity of hyperacusis. The THS is a nonvalidated instrument that is used to rapidly determine how much of a reported problem is due to tinnitus, hearing, and/or hyperacusis. This measurement is often necessary because tinnitus patients tend to confuse hearing problems with tinnitus problem. Therefore, the THS is an efficient screening tool that allows clinicians to differentiate which of the three problems (tinnitus, hearing, and/or hyperacusis) is most troublesome to the patient [37, 38]. A total score of 3 or more on the tinnitus portion of the survey may suggest a need for clinical intervention [38–41].

### 2.5. Tinnitus Problem Checklist (TPC)

The TPC is used to identify bothersome tinnitus situations [38]. The patient is instructed to select a situation where tinnitus is most bothersome. The patient then chooses the first, second, and third most bothersome tinnitus situations. The selections include falling asleep at night, staying asleep at night, waking up in the morning, reading, working on the computer, relaxing in my recliner, napping during the day, planning activities, driving, and others (where the patient can report a situation that is not listed among the choices).

### 2.6. Statistics

Analyses of continuous variables were performed using 2-tailed, unequal variance, Student's* t*-test, while analyses of nominal and ordinal variables were performed using Fisher's exact test. Fisher's exact test was chosen in place of Pearson's* chi*-squared test in order to eliminate limitations dealing with low counts of “yes” or “no” for some variables. Analysis of variance (ANOVA) was used to compare more than 2 groups of continuous variables. Linear regression was performed using Minitab 17. Odds ratios calculated from coefficients of the linear regression model are reported with 95% confidence intervals (CI). Other analyses were performed using Microsoft Excel 2010. Means are reported with ± standard deviations (SD). For all variables, a* P* value < 0.05 was considered to be statistically significant.

## 3. Results

Demographic, otologic, audiologic, and psychiatric profiles are presented in Table 1. Of 94 veterans, 93 (99% patients) were male and 1 was female. The mean age was 62 years. Veterans from the Army, Navy, and Air Force were represented. Military noise was the predominant source of noise exposure, affecting 67 (71%) patients. Vertigo was the most common otologic symptom, affecting 23 (24%) patients. The majority of veterans (90, 96%) had some degree of hearing loss in one ear or the other, and most of them (88, 94%) were diagnosed with sensorineural hearing loss. Sleep disorder, based on ICD-9 diagnoses by Behavioral Health Medicine, was found in 37 (39%) veterans. Furthermore, posttraumatic stress disorder (PTSD) was found in 77 (82%) patients, anxiety in 75 (80%) patients, and depression in 57 (61%) patients.

### 3.1. Tinnitus Characteristics

Features specific to tinnitus are presented in Table 2. Most veterans (74, 79%) had bilateral rather than monaural tinnitus, which was predominantly constant (versus intermittent) in 56 (60%) patients. More patients (20, 21%) began suffering from tinnitus within the last 1 to 10 years compared to any other time point. The most bothersome tinnitus situation was falling asleep at night (44 patients, 47%), followed by staying asleep at night (5 patients, 5%) and waking up in the morning (2 patients, 2%).

The average total THI score was 57 ± 23.5 (median 59, range 4–96), suggesting that this population of veterans fell within the severe level (score > 56) of tinnitus handicap and should undergo clinical intervention. The mean score on the tinnitus portion of the THS was 10 ± 4.0 (median 10.5, range 0–16), also indicating that this population had clinically significant tinnitus. The mean THS hearing score was 11 ± 4.5 (median 11.5, range 0–16), signifying the presence of concurrent hearing loss. The mean THS hyperacusis score was 1.9 ± 1.6 (median 2, range 0–4), implying a moderate problem with hyperacusis as well. The mean ESS score was 10 ± 5.4 (median 9, range 1–22), lying at the border of normal and abnormal daytime sleepiness. Thirty-eight (42%) patients had an ESS score of 10 or more.

### 3.2. The Epworth Sleepiness Scale as a Measure of Sleep Disturbance

Of those veterans with sleep disorder, the average ESS score was 15.4 ± 3.6, compared to 6.0 ± 2.2 for those without sleep disorder (*P* < 0.001). Using an ESS score of 10 as a cutoff for clinical significance, all patients were divided into 2 groups: ESS score < 10 and ESS score ≥ 10. Sleep disorder was found in 0 of 53 patients in the former group and 37 of 38 (97%) patients in the latter (*P* < 0.001). The positive predictive value (PPV) of the ESS for sleep disorder given the above cutoff was 97%, and the negative predictive value (NPV) was 100%. Thus, we elected to use the ESS score as a measure of sleep disturbance in the subsequent analyses.

### 3.3. Differences in Sleep Disturbance and Other Characteristics among Tinnitus Severity Groups

Based on the THI total score, veterans were divided into 4 tinnitus handicap severity groups: no tinnitus handicap (0–16), mild (18–36), moderate (38–56), and severe (>56). The no tinnitus handicap group had a mean ESS of 5.8 ± 2.9, the mild group 7.8 ± 1.6, the moderate group 10.1 ± 4.3, and the severe group 10.7 ± 6.3. ANOVA testing was performed to assess whether there was a difference in mean ESS score among the groups and no significant difference was found (*P* = 0.071). We proceeded with the analysis by aggregating the patients into fewer tinnitus handicap severity groups.

**Figure 1:**
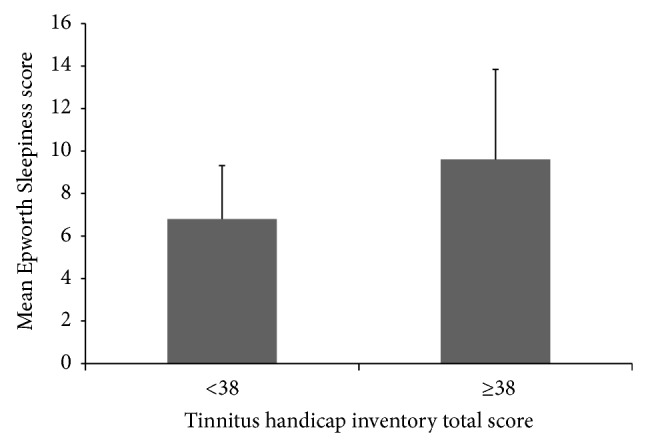
Mean Epworth Sleepiness Scale (ESS) score for Tinnitus Handicap Inventory (THI) total score <38 and ≥38. Vertical bars with caps indicate standard deviations.

Since a THI total score of 38 was suggested as the cutoff for clinical significance, we divided the patients into 2 groups: THI total score <38 and ≥38. The former group had a mean ESS score of 6.8 and the latter a mean of 9.6 (*P* = 0.006). This is shown in Figure 1. Subsequently, we searched for a difference between the 2 tinnitus groups with respect to each variable listed in Tables 1 and 2. The group with THI total score <38 was found to have a significantly lower incidence of PTSD, anxiety, and sleep disorder, higher average right ear word discrimination score, and lower THS tinnitus, hearing, and hyperacusis scores. Notably, there was no difference in other measures of hearing loss, and there was a near-significant difference in rate of depression (39% in THI <38 group, 67% in THI ≥ 38 group, *P* = 0.057).  Table 3 summarizes these significant factors.

**Figure 2:**
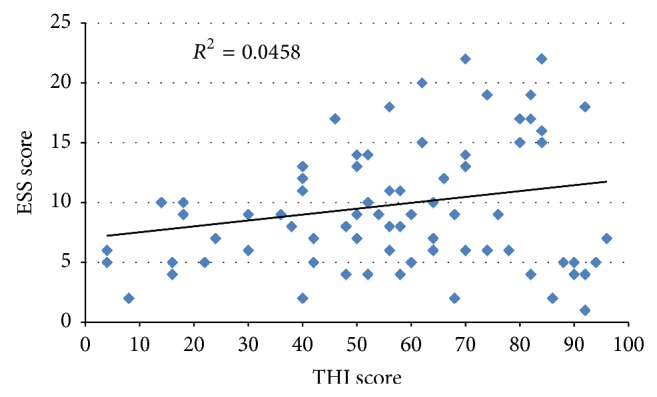
Scatterplot of Tinnitus Handicap Inventory (THI) score versus Epworth Sleepiness Scale (ESS) score.

A scatterplot was constructed to evaluate the global association between tinnitus (total THI score) and sleep disturbance (ESS score), as shown in Figure 2. On visual inspection, a portion of the data appeared to drift upwards while a portion appeared to plateau or drift slightly downwards at around an ESS score of 10. Taking this observation into account, the data was partitioned into 3 groups to more clearly define correlational attributes between these variables: patients with THI scores ≤46, designated as “normal”; patients with THI scores >46 and ESS scores ≥ 10, designated as “high-high”; and patients with THI scores >46 and ESS scores < 10, designated as “high-normal.” Table 4 provides a summary of correlation coefficients (*R* ^2^). Comparing the high-high group and the high-normal group in terms of characteristics listed in Tables 1 and 2, the former had a higher percentage of bilateral tinnitus (100% versus 80%, *P* = 0.025), lower left ear word discrimination score (89.8 versus 93.8, *P* = 0.049), and higher THS hearing score (13.1 versus 11.4, *P* = 0.028).

### 3.4. Predictors of Sleep Disturbance among Tinnitus Patients

To reveal which demographic, otologic, audiologic, or psychiatric variables are associated with sleep disturbance in tinnitus patients, we performed linear regression using all factors listed in Tables 1 and 2 (except sleep disorder) as independent variables to predict sleep disturbance in terms of ESS score < 10 or ≥10. Three variables were found to be significantly associated with an ESS score of ≥10: bilateral tinnitus (*P* = 0.006), vertigo (*P* = 0.036), and anxiety (*P* = 0.007). The odds ratios were 1.80 (95% CI 1.20, 2.70), 1.30 (95% CI 1.02, 1.66), and 1.62 (95% CI 1.15, 2.27), respectively. The adjusted correlation coefficient (*R* ^2^) was 0.223.

## 4. Discussion

Noise, or a perception thereof, can be a natural obstacle to resting and initiating sleep. Many studies have examined the relationship between tinnitus and sleep. An Italian group found that 54% of study patients had sleep disorders and that maintaining sleep was the predominant complaint [22]. Lasisi and Gureje studied 1302 elderly subjects and found that 11.4% had tinnitus, with 51.9% of those with tinnitus reporting insomnia, compared to only 33.8% of those without tinnitus (*P* = 0.002) [16]. In the same study, it was found that difficulty falling asleep and morning wakefulness were significantly associated with tinnitus. Likewise, a large cross-sectional survey of 4,705 tinnitus patients conducted in Germany discovered that nearly 77% had sleep difficulty and 46.4% attributed the cause of their sleep disturbance to tinnitus [6].

Clinical studies have linked tinnitus specifically to decreased sleep quality [6, 15, 19, 20]. Alster et al. used the Mini Sleep Questionnaire to compare 80 tinnitus patients and a control group (both groups representing military personnel without major psychiatric comorbidities), to find that 77% of the tinnitus group had worse scores, particularly in prolonged sleep latency, microarousals, and morning fatigue [19]. Attanasio et al. reported that sleep quality decreased with an increase in self-reported tinnitus severity and that there was a significant alteration in all stages of sleep in tinnitus patients, with greater periods of stage 1 and 2 sleep than stages 3, 4, and REM sleep [42]. Similarly, other studies found lower spectral power in the delta frequency band that appears in sleep stages 3 and 4 of tinnitus patients, which correlated with subjective sleep complaints [43, 44].

Despite the lack of a proven mechanism by which tinnitus directly causes sleep disturbance, some theories have been formulated. Tinnitus may interfere with sleep by the amplification of internally perceived noise during times of low environmental noise. Also, increased awareness of tinnitus before falling asleep may lead to increased focus on tinnitus and undue anxiety which may lengthen sleep onset latency [6, 22]. On a physiological level, tinnitus has been suggested to lead to sleep disturbance through cortical pathways involving the auditory cortex as well as other nuclei. In the gerbil animal model, tinnitus was induced by salicylate injection or loud noise exposure, and c-fos, a marker of neuronal activity, was screened for and found to be consistently expressed in the auditory cortex, the frontal cortex, areas responsible for behavioral and physiological stress reactions, and areas controlling autonomic function [45]. A similar pattern of c-fos expression was found in the brains of rats that were exposed to a stressor which led to increased sleep latency, decreased nonrapid eye movement (NREM) and REM sleep, and increased sleep fragmentation [46]. These and other animal studies have led to the notion that auditory information enters the amygdala from either the auditory cortex or the thalamic auditory relay nucleus and is thought to be enhanced in people with tinnitus. In doing so, areas of the amygdala are activated depending on the emotional significance of the stimuli, thereby eliciting a defensive response associated with fear [6].

Patients treated at Veterans Affairs (VA) Hospitals are particularly susceptible to tinnitus and its consequences. Veterans are exposed to high level of occupational noise, with increasing duration of military service correlating with lower hearing sensitivity [47]. Increased noise exposure is the most common cause of tinnitus [48]. As such, it was estimated in 2004 that 3 to 4 million American veterans were suffering from tinnitus and that tinnitus is regarded as one of the most disabling conditions caused by military service [49]. Its predominantly male sex and increased age make the VA population more vulnerable to tinnitus [24]. When the VA population is divided into 5-year age intervals, the greatest number of veterans lies between the ages of 65 and 69 [24]. Subjective tinnitus annoyance seems to increase in men with age, though this may partially be attributed to increased hearing problems with age [50]. For those older than 50 years, the likelihood of problems with sleep maintenance doubles [6].

Thirty-nine percent of our veteran population had an ICD-9 diagnosis of sleep disorder. This is within the range reported in the literature, as discussed previously (about 25–77%). An ESS score of 10 or above has been used as a cutoff for clinically abnormal daytime sleepiness. We found that, with this cutoff, the ESS was able to predict the presence of sleep disorder with a positive predictive value of 97% and negative predictive value of 100%. Therefore, the ESS can be an accurate tool for assessing the presence of sleep disturbance in veterans with tinnitus.

When veterans were divided into 4 tinnitus handicap severity groups, from no tinnitus handicap to severe, we did not find a significant difference in mean ESS score among the groups. However, the no tinnitus handicap and mild tinnitus groups had “normal” range (<10) (mean ESS scores 5.8 and 7.8, resp.), while the moderate and severe groups showed “abnormal” range (≥10) (ESS scores 10.1 and 10.7, resp.). Given the positive trend, there were possibly an insufficient number of subjects to detect a small difference in ESS scores. However, we were able to find a significant difference in ESS scores when patients were divided into 2 larger tinnitus handicap severity groups: THI < 38 and THI ≥ 38. We explored significant differences between these 2 groups and found that the THI < 38 group had a lower incidence of PTSD (59% versus 92%), anxiety (41% versus 91%), and sleep disorder (12% versus 47%). Fagelson found that the incidence of PTSD was 34% in a veteran population suffering from tinnitus, compared to our finding of 82% overall [51]. In primary care clinics, 5–13% of veterans have been found to have PTSD [52]. The general veteran population has been reported to have a lifetime PTSD prevalence of 31% in males and 27% in females [53]. Not only did our veteran population have a much higher rate of PTSD, but we also encountered a dramatic increase in PTSD with an increase in tinnitus handicap severity. This suggests that there is a positive linear or perhaps even exponential relationship between PTSD and tinnitus. Furthermore, there may be a synergistic effect involving tinnitus, PTSD, and sleep disorder in which the disease entities worsen one another's impact. Anxiety, which is closely related to PTSD, likely has a similar influence. Of note was the lower incidence of depression in the THI < 38 group compared to the THI ≥ 38 group (39% versus 67%). We suspect that depression also influences tinnitus, but perhaps our population was not large enough to capture the effect.

Interestingly, we discovered three distinct groups of patients as a function of THI and ESS scores. The group with THI total score ≤46 shared approximately 33% of the variance between THI and ESS scores. Above a score of 46, there was a group with increasing tinnitus handicap as well as increasing daytime sleepiness and a group with increasing tinnitus handicap and steady or decreasing daytime sleepiness. Coincidentally, the ESS cutoff score between the 2 groups approximately corresponds to the borderline of where sleepiness becomes clinically significant [28]. Previous documentation of this phenomenon was not found in the literature. The high-high group (THI > 46 and ESS ≥ 10) had a significantly higher percentage of bilateral tinnitus, lower left ear word discrimination score, and higher THS hearing score. No specific evidence relating tinnitus laterality to sleep disturbance was found in the literature. One can only speculate that unilateral tinnitus can be partially offset by head positioning on the pillow during sleep initiation. The low left ear word discrimination score combined with high THS hearing score might suggest retrocochlear involvement, but, in the absence of specific site-of-lesion assessments, such notions remain speculative.

Finally, we wanted to determine which factors may be used to predict sleep disturbance in a veteran population suffering from tinnitus. We found that bilateral tinnitus, vertigo, and anxiety were associated with ESS score ≥ 10. There is some evidence that lack of sleep may trigger migraines and migrainous vertigo, but no literature was found on a direct relationship between vertigo and sleep disturbance in tinnitus patients [54]. Anxiety has been well documented as a contributor to sleep disturbance [55, 56]. It has also been reported to correlate with tinnitus handicap severity [57].

It appears that PTSD, anxiety, and depression all exacerbate, to some degree, sleep disturbance and tinnitus in the veteran population. There are likely other psychiatric comorbidities that also play a role but were not studied in this paper. This underscores the importance of treatment for these disorders for patients with tinnitus. Antidepressants, such as selective serotonin reuptake inhibitors (SSRIs), have been shown to be effective in psychiatric disorders such as PTSD and depression [58, 59]. There is also evidence for sleep improvement when they are effectively used [60]. However, a 2006 Cochrane Review showed insufficient evidence that antidepressants improve tinnitus [61]. Perhaps future prospective studies can focus on the efficacy of antidepressants in veterans who are prone to, and present with, both psychiatric disorders and tinnitus, along with sleep disorder. Another avenue for research would be to compare the efficacy of psychotherapy versus antidepressants. What is the cause and effect relationship among tinnitus, sleep, and psychiatric comorbidities? The fact that many patients present with these disorders simultaneously, the lack of objective measurements for severity, and overlapping disease mechanisms all contribute to the complexity of this question. Future physiological studies using more objective tools such as polysomnography, brain MRI, and other imaging modalities, and molecular markers may better elucidate the relationship.

## 5. Conclusions

The ESS can be used as a tool in the initial assessment of sleep disorders in veterans with tinnitus, with a PPV of 97% and NPV of 100%. There is a positive correlation between tinnitus and sleep disturbance in veterans, as has been shown in the general populations. However, we discovered a higher rate of PTSD (82%) in veterans with tinnitus than previously reported in the literature and found that veterans may suffer from comorbidities such as PTSD, anxiety, and depression which appear to act synergistically to worsen sleep disturbance and/or tinnitus. There appears to be a distinct population of veterans who have high tinnitus handicap severity, yet no sleep disturbance. Unlike this group, those with high tinnitus handicap severity and abnormal sleep perceive increased hearing loss despite a lack of audiometric evidence, the reason for which is unclear. Furthermore, vertigo and bilateral tinnitus seem to contribute to sleep disturbance in tinnitus patients, the mechanism of which has not been explored. Our study supports a need for multifaceted management of tinnitus in veterans, including treatment for psychiatric comorbidities and sleep disturbance, in order to achieve an improvement in tinnitus handicap severity.

## Figures and Tables

**Table 1:** Demographic, otologic, audiologic, and psychiatric profiles. SD: standard deviation, normal ear: tympanic membrane and external auditory canal, 3F-PTA: 3-frequency-pure-tone average (500, 1,000, and 2,000 Hz), SRT: speech reception threshold, WDS: word discrimination score, and PTSD: posttraumatic stress disorder.

Total of 94 patients	Patients	% (mean ± SD, median, min–max)	Unknown
Sex			
Male	93	99	0
Female	1	1	0
Age		(62 ± 10.3, 64, 31–84)	
Service			
Army	35		41
Navy	13		
Air force	5		
Noise exposure			
Military	67		24
Occupational	36		25
Recreational	21		39
All	69		21
Previous hearing aids use	18		7
Vertigo	23		20
Middle ear symptoms	14		9
Ear surgery	7		2
Ear injury	9		23
Ear infection	10		45
Ear pain	16		36
Aural fullness or pressure	18		32
Normal ear			
Left	91	97	0
Right	90	96	0
Left hearing loss			
Sensorineural	87	93	2
Mixed	2	2	
Right hearing loss			
Sensorineural	86	91	2
Mixed	2	2	
3F-PTA			
Left	94	(28 ± 14.3, 25, 6.7–83.3)	0
Right	94	(27 ± 15.3, 25, 5–93)	0
SRT			
Left	92	(25 ± 13.5, 25, 0–80)	2
Right	91	(24 ± 15.1, 20, 0–100)	3
WDS			
Left	92	(91 ± 12.7, 95, 0–100)	2
Right	92	(90 ± 15.1, 96, 20–100)	2
Sleep disorder	37	39	3
PTSD	77	82	4
Anxiety	75	80	2
Depression	57	61	1
Claustrophobia	9		48

**Table 2:** Tinnitus characteristics.

Total of 94 patients	Patients	% (mean ± SD, median, min–max)	Unknown
Tinnitus quality			
Bilateral	74		13
Unilateral	7		
Constant	56		14
Intermittent	24		
Tinnitus duration			
≤1 year	3		37
2–10 years	20		
11–20 years	4		
21–30 years	17		
31–40 years	5		
41–50 years	7		
>50 years	1		
Tinnitus Handicap Inventory			
Total score	94	(57 ± 23.5, 59, 4–96)	0
Functional score	94	(29 ± 11.4, 31, 0–44)	0
Emotional score	94	(17 ± 9.1, 18, 0–32)	0
Catastrophic score	94	(11 ± 4.8, 10, 2–20)	0
Tinnitus and Hearing Survey			
Tinnitus score	94	(10 ± 4.0, 10.5, 0–16)	0
Hearing score	94	(11 ± 4.5, 11.5, 0–16)	0
Hyperacusis score	94	(1.9 ± 1.6, 2, 0–4)	0
Tinnitus Problem Checklist: most bothersome situation			
Falling asleep at night	44		20
Staying sleep	5		
Waking up in the morning	2		
Napping during the day	0		
Epworth Sleepiness Scale score	91	(10 ± 5.4, 9, 1–22)	3

**Table 3:** Significant differences between tinnitus severity groups. THI: Tinnitus Handicap Inventory, SD: standard deviation, PTSD: posttraumatic stress disorder, R WDS: right ear word discrimination score, and THS: Tinnitus and Hearing Survey.

Factor	THI < 38		THI ≥ 38		*P* value
	Incidence	Mean ± SD	Incidence	Mean ± SD	
PTSD	58.80%		91.80%		0.002
Anxiety	41.20%		90.70%		<0.001
Sleep disorder	11.80%		47.30%		0.007
R WDS		96.0 ± 4.2		89.2 ± 16.5	0.002
THS score					
Tinnitus		4.5 ± 3.1		11.3 ± 3.0	<0.001
Hearing		6.5 ± 5.2		11.7 ± 3.7	0.001
Hyperacusis		0.9 ± 1.0		2.2 ± 1.6	<0.001

**Table 4:** Correlation coefficients for Tinnitus Handicap Inventory (THI) total score and Epworth Sleepiness Scale score.

Group	*R* ^2^
All patients	0.046
THI ≤ 46	0.332
THI > 46 and ESS ≥ 10	0.271
THI > 46 and ESS < 10	0.213
